# Adverse event detection by medical record review is reproducible, but the assessment of their preventability is not

**DOI:** 10.1371/journal.pone.0208087

**Published:** 2018-11-29

**Authors:** Dorthe O. Klein, Roger J. M. W. Rennenberg, Richard P. Koopmans, Martin H. Prins

**Affiliations:** 1 Department of Clinical Epidemiology and Medical Technology Assessment (KEMTA), Maastricht University Medical Centre+, Maastricht, the Netherlands; 2 Department of Internal Medicine, Maastricht University Medical Centre+, Maastricht, The Netherlands; 3 Department of Epidemiology, School for Public Health and Primary Care, Maastricht University, Maastricht, the Netherlands; Institute of Tropical Medicine Antwerp, BELGIUM

## Abstract

**Objective:**

To assess the reproducibility of adverse event evaluation by a medical record review committee.

**Design:**

Cross-sectional reanalysis of medical records.

**Intervention:**

Reviewers re-examined fifty medical records of deceased patients regarding the presence of adverse events, their potential preventability and their possible contribution to death. Also we investigated the root causes of the preventable AEs. Differences between the first and second assessment were calculated.

**Results:**

The Kappa on the presence of an adverse event was 0.64 and 0.32 for the potential preventability. The intrarater agreement showed a Kappa of 0.61 on the adverse event presence and 0.64 for the potential preventability. Interrater agreement showed a Kappa of 0.66 for the adverse event presence and 0.03 for the potential preventability.

**Conclusion:**

We found a fair reproducibility for the detection of adverse events, but a poor reproducibility for the potential preventability. Possibly this was caused by lack of a definition for the preventability of adverse events. We think giving feedback to professionals using the results of medical record review remains valuable, but an improvement of its reproducibility is essential. To our opinion an international consensus on what exactly constitutes preventability of adverse events and agreement on a definition is necessary. This would result in more comparable studies in this field and could then be more informative on the ideal procedure to avoid certain potentially preventable adverse events in the future.

## Introduction

The quality and safety of patient care have gained attention in the past decades. Many methods are used with the aim to improve the quality of care. One of these methods is medical record review.

In several university hospitals in the Netherlands the focus of medical record review is on the detection of preventable adverse events (AEs) in patients who have died during their stay. The records of these group of patients are assumed to contain more (preventable) AEs in comparison with discharged (alive) patients.[1]

Often, trigger systems are used for medical record review because these lower the burden of review, by selecting records. Triggers are clues to alert the screeners for potential AEs so the medical record can be reviewed to determine if an actual AE has occurred.[2–5] Nonetheless, the conclusion that a potentially preventable AE has occurred is stressful for the involved professionals. A common reaction is therefore to dispute the judgment of the committee instead of evaluating the case itself and subsequently improving care.[6, 7] To prevent discussions regarding the accuracy of the judgment post hoc, this judgment should be reliable and reproducible.[8–13]

It is likely that if the committee judgment would prove to be reproducible, professionals are more inclined to accept the outcome. There are several studies which investigated the inter-rater reliability using the Harvard medical practice study (HMPS) trigger tool regarding the presence and the preventability of an AE in a mixed (alive and deceased) population. However, these studies showed a moderate reliability for the presence and preventability of an AE.[14–29] Moreover, improving quality and safety of care in an already reasonable safe environment leads to an exponential increase in costs. Therefore, we think health care providers should select the most optimal tests to measure and improve their performance. In order to determine the best methods, qualifying them, making them comparable, and optimising their use, further research is necessary. In our search for better, more reliable methods we hypothesized that our method would show better outcomes compared to previous studies because in our centre the entire review committee discusses the results of the medical record evaluation instead of in pairs or by oneself as is usually reported.[4, 30–32]

Therefore, our main question in this study was: ‘‘What is the reproducibility of the judgment of medical records by an internal committee reviewing medical records of deceased patients regarding AE presence. Moreover, we also evaluate the root cause of AEs and their reproducibility of these AEs concerning their preventability and their contribution to death?”

## Methods

### Medical record review method

Since 2008, a stable team of experienced nurses (each with more than 10 years of clinical experience) with an affinity for record review and patient safety, screen the medical records of all deceased patients for the presence of triggers. Medical record review was introduced in our hospital in 2008 as a project called ‘‘preventing medical injury.” The aim of this project was to improve the quality and safety of patient care by reducing and prevention of unintended medical injury happening to patients in our hospital.

**Fig 1:**
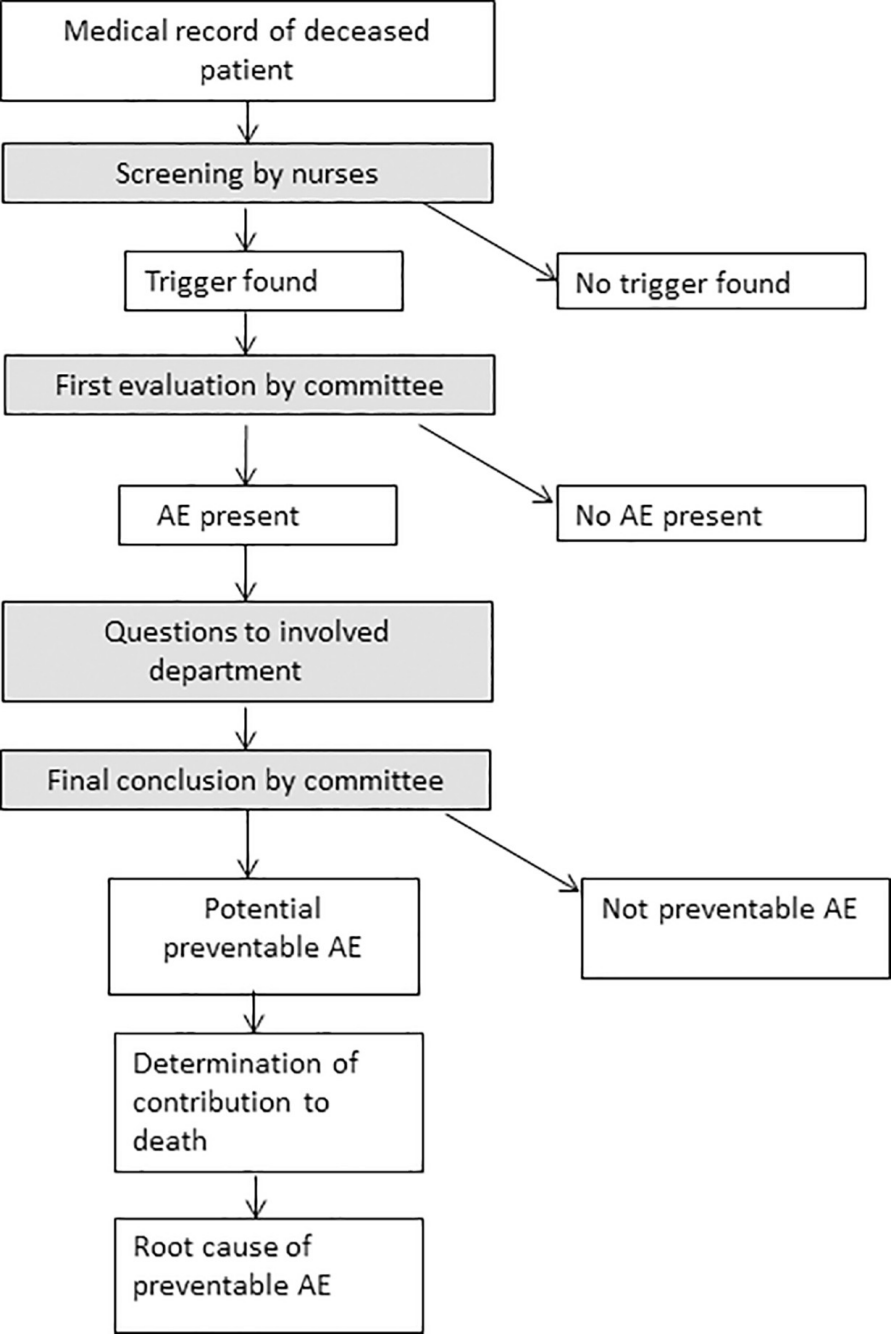
Flowchart of the medical record review procedure.

We use a slightly adapted version of the HMPS trigger system[16] to make it suitable for the screening of deceased patients. Therefore, triggers regarding transfer to another acute care hospital and unplanned inappropriate discharge to home were omitted, as they have no relevance in deceased patients. In case a medical record has at least one trigger, it is forwarded to the review committee. Further explanation on the trigger system is published in larger detail elsewhere.[33] The procedure of the medical record review is also shown in Fig 1. The study protocol was approved by the medical ethics committee of our hospital. The committee explicitly gave their oral consent to participate in this study and publication of the results.

### Definitions

The following definitions were applied in the medical record review:

An AE was defined as an unintended outcome caused by the (non-)action of a caregiver and/ or the healthcare system resulting in temporary or permanent disability or death of the patient. An AE was considered as preventable, if in retrospect after a systematic analysis of the events, it seems that certain measures could have resulted in the prevention of the AE.[34]

### Committee

The medical records with a trigger which are forwarded to the review committee, are redirected to the member with experience and expertise concerning the case. For example, if a patient died during a surgical procedure, a surgeon analyses the medical record for AEs. We chose this approach because we believe that the considerations on the presence of an AE are best made by a specialist experienced with the usual procedures, protocols and the possible treatments for a certain disease. The committee consists of 9 specialists with different specialties (internist, neurologist, pulmonologist, pediatrician/neonatologist, internist/oncologist, anesthetist, surgeon, cardiologist, cardiothoracic surgeon and internist/geriatrician) representing the departments with most of the in-hospital deaths. The members are both active (working in our hospital) and recently retired (who used to work in our hospital) experienced specialists.

After a thorough investigation by this committee member, the results are presented to the rest of the committee. A first conclusion on the potential presence of an AE is established in a weekly meeting with at least 4 committee members present. Subsequently, after consulting the involved physicians, the committee finally decides on the presence of an AE, the potential preventability, and the possible contribution of this AE to the death of the patient. If an AE was considered potentially preventable the committee identified the preventable cause according to a standard list of factors (S1 Appendix).

### Data

The results of the triggering by the nurses and the review by the specialists are saved using software provided by Medirede, Clinical File Search version 3 (Mediround BV, 2015). This software was designed to store the data of the medical record review in a clear and easily accessible way.

### Selection of records for this study

**Fig 2:**
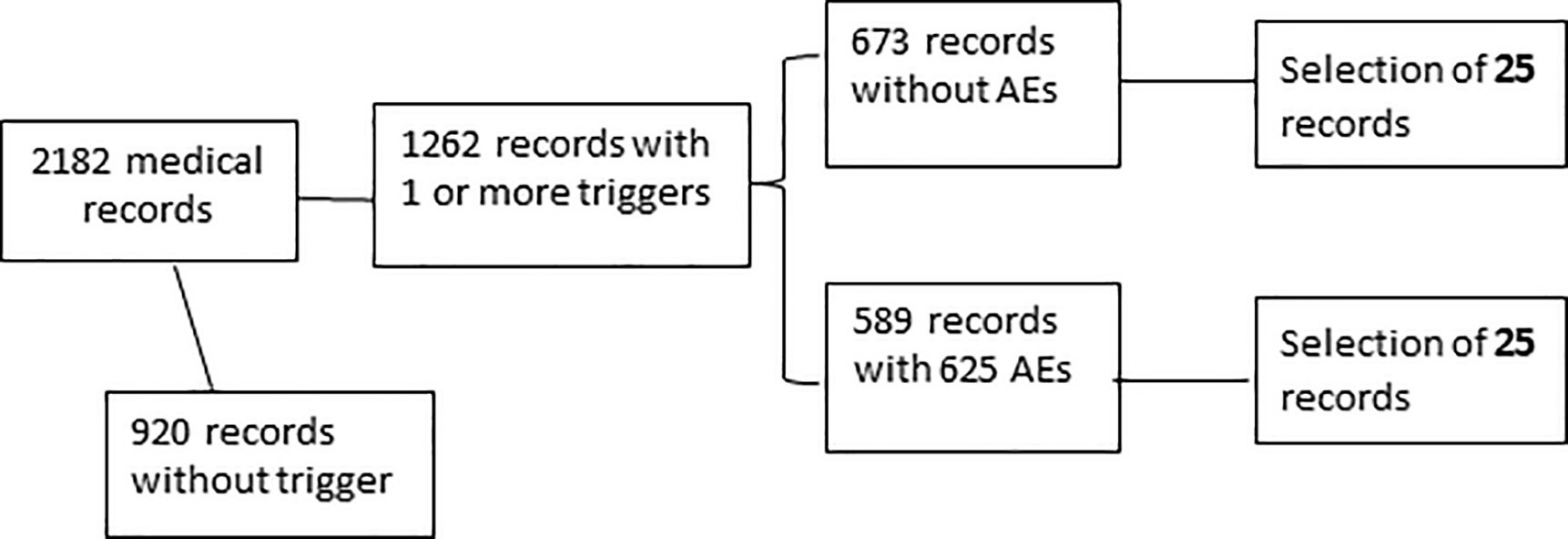
Flowchart of the selection of the medical records in this study.

With regard to our main research question we aimed to get a point estimate with a 95% confidence interval of 6% to each side. Hence, we needed to include 50 cases for the evaluation of the reproducibility of the AE. We randomly selected these medical records out of the database which contained 1262 previously triggered and evaluated medical records of deceased patients between 2013 and 2015. This period was chosen to avoid the presence of recently debated cases in the study sample and therefore minimizing the risk of recognition of cases and remembering former decisions by the committee members. Fig 2 shows the random selection procedure. We used the Excel random generator for the random selection of the study records. Of the 50 randomly selected cases, 25 had an AE after the first evaluation by the committee. We blinded the committee for the previous results of their medical record review. Therefore, the digital results of the first screening round were inaccessible and thus irretrievable for the reviewers.

### Analysis

After this re-evaluation of the medical records by the committee, we compared the outcomes of the two rounds regarding the presence of AEs, the preventability and their contribution to the death of the patient on a team level. The preventability of an AE was scored as being potentially preventable or not preventable. The contribution of an AE to the death of the patient was also scored dichotomous as potential contributing to death or no contribution.

Hence, we calculated the observed overall agreement (in %) to get an impression of the reproducibility of the judgment. By using cross tabulation, we calculated the observed overall agreement (accuracy) within the three groups (presence, preventability, and contribution) with the corresponding 95% confidence interval. Therefore, the number of both rounds negative (without AE) and both rounds positive (with AE) was summed and divided by the total number of medical records. Furthermore, we calculated the positive and negative agreement separately. To determine the reliability for AE presence, preventability, and contribution to death, we executed Cohen’s Kappa statistics and the McNemar’s test. A p-value of <0.05 was considered statistically significant. Because the judgment of the committee might be influenced by the presenter of a case, we also evaluated the outcomes of the medical record review after the review and presentation to the committee by different specialists (inter-rater reliability) and by the same specialist (intra-rater reliability). Thereafter, we checked if the AEs found in the second evaluation were the same as the original ones found. Finally, we compared the preventable causes to determine agreement between the first and second evaluation.

## Results

### Analyses comparing the outcomes of the first, and the second round on a team level

#### Presence of an AE (Table 1)

During the first screening of the medical records (previous to the study), the committee found 25 AEs, the second time (during the study) 28 AEs were found. There was an overlap of 22 AEs which were found both times. The observed agreement for the presence or absence of an AE was 82.0%. The corresponding Cohen’s Kappa was 0.64 (95%CI 0.48–0.80). McNemar’s test showed a value of 0.51, this means there was no significant difference in the proportion of AEs found in both rounds.

**Table 1:** AEs found in first and second round.

		Second round		Total
		Absent	Present	
**First round**	Absent	19	6	25
	Present	3	22	25
Total		22	28	50

#### Presence of potential preventability

In the first round, 17 of the 25 AEs had the indication to be potentially preventable, in the second round 11 of the 28 AEs were found to be potentially preventable. The corresponding observed overall agreement of the preventable AES was 65% (Table 2), and the Cohen’s Kappa was 0.32 (95%CI 0–0.65). McNemar’s test showed a value of 0.070, which indicates there was no significant difference in the proportion of preventability found in both rounds.

**Table 2:** Potential preventability of the AEs found.

		Second round		Total
		Not preventable	Potentially preventable	
**First round**	Not preventable	5	1	6
	Potentially preventable	7	10	17
Total		12	11	23

#### Possible contribution to death of the patient

For the calculation of the agreement regarding the possible contribution of the AE to the death of the patient, we compared the cases (22) with the same AEs in both rounds. 21 of these AEs were considered to possibly have contributed to the death of the patient, in the first round. (McNemar = 0.125) During the second screening, the committee concluded that 22 of the 22 AEs possibly contributed to the death of the patient. The accuracy was, therefore, 95% (Table 3).

**Table 3:** Potential contribution to death in both rounds.

		Second round		Total
		No contribution	Potential contribution	
**First round**	No contribution	0	1	1
	Potential contribution	0	21	21
Total		0	22	22

### Intrarater reliability–repeatability (individual level)

31 of the 50 cases were reviewed by the same committee member during the evaluation in both rounds. The observed overall agreement for the presence of an AE in this subgroup was 81%, and the corresponding Kappa was 0.61 (95%CI 0.33–0.89). The agreement on the presence of an AE was 79% and the agreement on the absence of an AE was 82%. McNemar’s test showed a value of 1.000, thus there was no significant difference found in the proportion of AEs in both rounds. For the preventability of the AEs the observed overall agreement was 57%, and the corresponding Kappa 0.64 (95%CI 0.18–1). The agreement on the presence of an AE was 100% and the agreement on the absence of an AE was 60%. An exact McNemar’s test determined that there was no significant difference in terms of the preventability of the AEs found the first and second time (p = 0.500). 11 cases in which a possible contribution to death was concluded in the first screening, the second screening found the same result, this means a 100% agreement.

### Interrater reliability–reproducibility (individual level)

19 of the 50 medical records were scrutinized by different doctors during their evaluation in both rounds. The observed overall agreement for the presence of an AE was 84%, and the Cohen’s Kappa was 0.66 (95%CI 0.30–1) in this subgroup. The agreement on the presence of an AE was 79% and the agreement on the absence of an AE was 100%. McNemar’s test showed a p-value of 0.250, no significant difference in the proportion of AEs found in both rounds. For the preventability, the observed overall agreement was 45%, and the Cohen’s Kappa was 0.03 (95%CI 0–0.55). The agreement on the presence of an AE was 75% and the agreement on the absence of an AE was 29%. An exact McNemar’s test determined that there was no significant difference in the proportion of preventability of the AEs found the first and second time (p = 0.219). In 10 out of the 11 cases in which a possible contribution to the death of the patient was determined in the first screening, the second screening concluded the same, resulting in 91% agreement.

### Root cause analysis

The total number of cases with a potentially preventable adverse event in both sessions, hence labeled with a suspected cause, was nine. The overall agreement on this cause was 78%, with a Kappa of 0.5 (95% CI 0–1). McNemar’s test was 1.00 indicating no significant difference.

## Discussion

This is the first study assessing the inter- and intrarater reliability for the presence of AEs, their preventability and potential contribution to death among deceased hospital patients. We showed that independent of the committee member involved in the second evaluation, there is a good reliability for the presence of an AE, but only a fair reliability regarding the preventability of an AE. Potential contribution to death was highly reproducible. However, it should be realized that this finding could be distorted by the small number of cases (11) with the same AE in both rounds which were considered to contribute to the death of the patient. Moreover, there was almost no AE that did not contribute to the death of a patient. At the same time, it was difficult to exclude that an AE did not contribute to the death of the patient. It seems, therefore, that the trigger system especially selects cases with AEs that contribute to or cause death. In addition, we couldn’t calculate the Kappa for this section due to the low numbers in the cells of the two by two tables.

Our committee found more AE’s when scrutinizing the same records for the second time. Therefore, the question remains if the number of AEs found eventually is the true number of AEs. Even if the inter-rater reliability would be acceptable, there is no evidence that this kind of record review really detects all AEs.[14]

A strong point of our study is, in contrast to most other studies, the fact that reviewers discussed the outcomes of the medical record analyses in a group session. In these other studies, usually pairs of reviewers perform the medical record review. A study by Hofer et al (2000) found that discussion between reviewers didn’t improve the overall reliability, with a Kappa of 0.36 before and a Kappa of 0.40 after discussion.[35] Interestingly, in our study where cases were discussed twice (during the first and second round), we found a substantially higher level of agreement with a Kappa of 0.64. However, the discussion in our study was between the same committee members both times, whereas in the study of Hofer et al, it was between different pairs of reviewers.

The results from our subgroup analyses suggest a rather low inter-rater reproducibility concerning the preventability of the AEs, in contrast to a higher intra-rater reproducibility. The same phenomenon is also seen in other studies in which both hospital survivors and deceased patients were included. [23–30] Although our system is different in some crucial parts, the judgement concerning the preventability of AEs is comparable with the results in these studies.

Several studies use different classifications for preventability. 3-level classification systems and also a 5- and 6- level classification systems are used in studies.[23, 36, 37] This makes comparisons between studies difficult. There is no gold standard concerning potential preventability which leaves us with the consensus of the medical record research committee as second best. Differences in opinion will therefore always exist, this might give rise to an ongoing discussion. Nevertheless, the effort to detect AEs and their preventability is in our opinion useful because any preventable cause that can be abolished is important, therefore adequate feedback, resulting in consecutive change of procedures if necessary, is needed.

Our study had some limitations. A few methodological aspects of our study require attention. We blinded our reviewers and used records from several years ago, which we consider as a strong point, although we cannot exclude that they may have been able to recognize cases from their memory. Furthermore, there is the lack of a clear definition (which is also not available in international literature) to determine preventability. We think this is the reason why the reliability of the judgment on preventability is disappointing. Although the committee judgment on preventability became based on more experience over the past years, there are no strict guidelines or rules on how to judge. Previous studies sometimes use Likert scales or percentages which in our opinion creates a false sense of precision, with inconsistent reproducibility.[13, 20, 38–42] Secondly, the sample size of the re-examined cases was rather small, especially in the subgroup analysis. This was reflected in the root cause analysis in which only nine cases remained to be analysed. Therefore, we were unable to answer all of our sub questions, especially the one focusing on the reproducibility of the contribution of the AE to the death of the patient. The small number of cases was the result of powering the study to our main question. Moreover, increasing the number of cases to answer our sub-questions with sufficient power would have been very time and hence cost consuming.

In conclusion, we showed that the judgment on the presence of an AE by a committee investigating medical records of deceased patients was reproducible. However, their judgment on preventability is less so, possibly because there is a lack of clear definitions on this subject. It is difficult to comment on the reliability of contribution to death because all AEs seem somehow to contribute to the death of the patient in both rounds. Despite this, we think giving feedback to professionals based on the review of preventable AEs is mandatory. Yet, we should focus on finding more reliable methods to identify preventable AEs. The next step should be, a clear international accepted definition on preventability which could aid to increase the reproducibility of these methods and thus make them more valuable. Thereafter, respectful communication with the involved professionals in order to improve the quality and safety of care is of utmost importance to increase the chance that care will really become better.

## Supporting information

S1 Appendix (DOCX)

S1 Data (XLSX)
