# Supplementary material for: Adverse event detection by medical record review is reproducible, but the assessment of their preventability is not
Source: PLoS One. 2018 Nov 29;13(11):e0208087. doi: 10.1371/journal.pone.0208087 (PMC6264838; doi:10.1371/journal.pone.0208087)
Supplement: S1 Appendix — (DOCX) [file pone.0208087.s001.docx]

**S1 Appendix**

*Table X Most important factors contributing to preventable AEs in MUMC+*

| Factors |
| --- |
| 1. 1) Diagnostics and other actions prior to treatment    1. Knowledge, skills, code of conduct    2. Wrong/missed diagnosis, under- or overdiagnosis and evaluation    3. Wrong indication (beneficial effect of the treatment doesn’t compensate for the burden of the treatment; no beneficial effect expected; less invasive treatment has an equal effect)    4. Estimation of the patient capacity/treatment burden incorrect    5. Other…. |
| 1. 2) Treatment   Knowledge, skills, interpersonal skills   - 1. Surgical technique (surgery, instrumental intervention)   2. Medication technical   3. Medication (wrong dosage, side effect)   4. Nursing   5. Other… |
| 1. 3) Follow-up process   Wrong assessment of the disease severity/symptoms, complications (missing gut-feeling resulting in no action or too late action/inadequate diagnostics)   - 1. Follow-up of the patient (inaccurate)   Follow-up of the patient (incompetent)   - 1. Other… |
| 1. 4) Communication, cooperation and reporting   Communication during transfer from one location to another   - 1. Communication during transfer between physicians/nurses/paramedics   Cooperation of healthcare providers   - 1. Inadequate reporting   2. Other…. |
| 1. 5) Organizational/technical defects   Protocols/procedures/organization  Equipment/materials   - 1. No medium care/ incorrect patient placement assignments   2. Other… |
| 1. 6) Disease-related factors    1. Compliance by the patient    2. Disease severity of the patient    3. Comorbidity    4. Other.. |
| 1. 7) Other |
